# Evaluation of the association between fear of COVID-19 and pregnancy distress

**DOI:** 10.4314/ahs.v23i1.8

**Published:** 2023-03

**Authors:** Rojjin Mamuk, Şahide Akbulut, Ayfer Erdoğan

**Affiliations:** 1 Nursing Department, Faculty of Health Sciences, Eastern Mediterranean University. Famagusta, North Cyprus; 2 Nursing Department, Faculty of Health Sciences, Batman University, Batman, Turkey; 3 Nursing Department, Sabahattin Zaim University, Istanbul, Turkey

**Keywords:** COVID-19, fear, distress, pregnant women, pregnancy

## Abstract

**Background:**

Mental health problems experienced during pregnancy negatively affect both maternal and fetal wellbeing.

**Objective:**

This study aimed to investigate the relationship between fear of COVID-19 and pregnancy distress in healthy pregnant women living in Turkey.

**Methods:**

A descriptive, relational/cross-sectional study was conducted by interviewing 363 pregnant women in person. Data were collected using a personal information form, the Fear of COVID-19 Scale (FCV-19S), and the Tilburg Pregnancy Distress Scale (TPDS).

**Results:**

The mean FCV-19S score was 19.03±5.65 and the mean TPDS score was 19.97±7.97. According to the TPDS cut-off score, 19.0% of the participants were at risk of pregnancy distress. There was a significant positive correlation between FCV-19S and TPDS scores (r = 0.263, p<0.05). According to the regression analysis, age (β= -0.217), years of education (β= -0.272), and number of births (β= 0.502) were associated with fear of COVID-19, and fear of COVID-19 was associated with TPDS scores (β= 0.369) (p<0.05).

**Conclusion:**

The pregnant women in this study had moderate fear of COVID-19. Compared to the literature data, the prevalence of pregnancy distress was slightly higher than pre-COVID-19 reports but quite low compared to other studies conducted during the pandemic.

## Introduction

Since the first case was reported in Wuhan, China, novel coronavirus disease 2019 (COVID-19) has become one of the biggest pandemics in human history, with 5.054.267 confirmed deaths worldwide^1^. The physiological and immunological changes that occur during pregnancy create a natural susceptibility to respiratory tract infections and severe pneumonia^2^. Therefore, pregnant people were included among the risk groups during the COVID-19 pandemic^3^. Studies examining the perinatal outcomes of COVID-19 have reported that in addition to those who are discharged without complications, there are also cases of preterm birth, low birth weight, increased preeclampsia and cesarean rates, and unfortunately, perinatal death. Although there is no evidence of vertical transmission of SARS-CoV-2, uncertainty is a concern both for science and for expectant parents^4^. As a result, pregnant people fear for their and their baby's health and experience moderate to high levels of stress^5^–^7^. However, stress can also lead to adverse perinatal outcomes such as premature birth and low birth weight6. This poses a double threat, as perinatal outcomes are affected both by actual COVID-19 infection and the fear/anxiety related to the disease. Other mental problems experienced by pregnant people due to COVID-19 include anxiety, depression, sleep disorders, and post-traumatic distress syndrome^8^–^14^. Even under normal circumstances, stress, depression, and/or anxiety can occur during pregnancy^15^. This condition is referred to as pregnancy distress and has been attributed to various factors, including pregnancy-related physical and social changes, medical problems, fear of childbirth, and efforts to adapt to parenthood^16^–^18^. The prevalence of pregnancy distress varied between 23% and 38% in pre-COVID-19 studies19,20. In studies conducted in Turkey, this figure ranged from 9% to 33% 21–23. In a Turkish study of at-risk pregnant women conducted during the COVID-19 pandemic, the prevalence of distress among pregnant women was reported to be 37% 24. In another study including healthy pregnant women, the prevalence rates of anxiety and depression were 64.5% and 56.3%, respectively 25. However, there are no studies investigating the relationship between fear of COVID-19 and pregnancy distress. Therefore, this study aimed to examine the relationship between fear of COVID-19 and pregnancy distress in healthy pregnant women living in Turkey.

## Research Questions

1. To what degree do pregnant women fear COVID-19?

2. What is the level of pregnancy distress in pregnant women during the COVID-19 pandemic?

3. Is there a relationship between pregnant women's fear of COVID-19 and pregnancy distress?

4. Are the socio-demographic and obstetric characteristics of pregnant women associated with their fear of COVID-19 and pregnancy distress?

## Methods

### Design and study setting

This descriptive/cross-sectional study was conducted between October 26 and December 30, 2020 in a women and children's hospital in Batman, Turkey. The Batman province is located in a generally rural area and ranks 70th among the 81 provinces of Turkey in terms of socioeconomic development 26. A traditional and patriarchal lifestyle predominates and there is a generally positive attitude towards childbirth in this province. Therefore, the total fertility rate in the province is among the highest in Turkey 27. The study setting was a public hospital that mostly serves low- and middle-income Turkish citizens, Syrian refugees, and migrants.

Starting in September 1, 2021, COVID-19 vaccines were administered to pregnant women in 34 countries, including the United Kingdom, Spain, Sweden, Switzerland, Norway, Finland, the United States, and Canada. In Turkey, vaccination started with healthcare workers on January 14, 2021, followed by individuals over 65 years of age and risk groups. Vaccination was started for individuals aged 50 years and older on May 31, 2021 and was gradually opened to lower age groups^28^. Pregnant women were included in the vaccination schedule according to these age groups^29^. Therefore, our study was conducted during a critical period before the start of COVID-19 vaccination in our country, including for pregnant women, and for this reason we could not evaluate the impact of vaccination on fear of COVID-19 and pregnancy distress.

### Study population and sample

Participants were recruited by convenience sampling, one of the improbability sampling methods. Inclusion criteria were being 18 years of age or older, being at 12 weeks of gestation or later, being able to understand and speak Turkish, and volunteering to participate in the study. Exclusion criteria were having a high-risk pregnancy, a diagnosis of any mental illness, or history of COVID-19 infection. The sample size for the study was calculated based on the expected prevalence in the population. A study conducted in Turkey was used as a reference for statistical power analysis^22^. The minimum number of participants required to estimate a prevalence of 33% with precision of 5% and confidence level of 95% was determined as 339. To reach the desired sample, 447 women were invited to complete a questionnaire, and all of these women constituted the study population. A total of 389 of the 447 invited women responded. Of these, 26 women were excluded because they did not provide complete questionnaire responses. As a result, the study was completed with 363 participants.

### Data Collection Process and Tools

Data were collected by conducting face-to-face interviews with the participants in a room in the antenatal outpatient clinic in accordance with protection measures against COVID-19. After obtaining their written and oral consent, we asked the participants to complete the questionnaire. We completed the questionnaire on the behalf of illiterate participants based on their self-report. Study data were collected using a personal information form, the Fear of COVID-19 Scale (FCV-19S), and the Tilburg Pregnancy Distress Scale (TPDS). It took the pregnant women approximately 10–15 min to fill in these forms.

### Personal Information Form

Based on our review of the literature, we designed this form to assess factors that may be associated with fear of COVID-19 and pregnancy distress 6,8,13,14. The form included questions about the participants' demographic characteristics (age, education, place of residence, economic status, employment status, presence of social support) and obstetric characteristics (weeks of gestation, trimester, number of pregnancies and births, history of miscarriage, and planned mode of delivery).

### Fear of COVID-19 Scale (FCV-19S)

The scale was developed by Ahorsu et al. (2020) to assess fear of COVID-19 30. It is a unidimensional instrument consisting of 7 items rated on a 5-point Likert-type scale (1 to 5 points) for a total score ranging from 7 to 35. There is no cut-off value; higher scores indicate a higher level of fear of COVID-19. Bakioğlu et al. performed the Turkish adaptation and validity study for the scale in 2020 and reported a Cronbach's alpha value of 0.88 31. In our study, we calculated a Cronbach's alpha value of 0.82.

### Tilburg Pregnancy Distress Scale (TPDS)

The TPDS was developed by Pop et al. (2011) to identify distress during pregnancy^15^. It consists of 16 items in two subscales (Negative Affect and Partner Involvement) and is applicable in women at 12 weeks of gestation or later. The Negative Affect subscale includes 11 items and yields a score between 0 and 33; the Partner Involvement subscale includes 5 items and yields a score between 0 and 15. Total TPDS scores range from 0 to 48. Çapık and Pasinlioğlu performed the Turkish adaptation and validity studies of the scale in 2015. For the Turkish version, they reported cut-off values of 28 for total score, 10 for the Partner Involvement subscale, and 22 for the Negative Affect subscale. Women with scores at or above these cut-off points are considered at risk of pregnancy distress. The Cronbach's alpha value of the Turkish version of the scale was reported to be 0.78 32. In this study, the Cronbach's alpha of the scale was 0.73.

### Statistical analyses

The data were analysed using IBM SPSS Statistics for Windows version 25.0 software (IBM Corp, Armonk, NY). Number, percentage, mean, and standard deviation were used as descriptive statistical methods. Kolmogorov-Smirnov test was used to determine whether the data were normally distributed. As the quantitative data showed normal distribution, we used independent samples t-test for comparisons between two independent groups and one-way analysis of variance with post hoc Bonferroni test for multiple comparisons. Pearson correlation analysis was used to evaluate the relationship between numerical variables. In addition, logistic regression analysis was performed to identify factors associated with fear of COVID-19 and pregnancy distress. The results were analysed within a 95% confidence interval and evaluated for significance based on an alpha value of 0.05.

### Ethics

Before starting the study, ethical approval was obtained from the Batman Education and Research Hospital Ethics Committee (Dated 22.10.2020, Decision no: 2020/5-4) and permission to conduct the study was obtained from the Ministry of Health and the relevant hospital. Written and verbal consent was obtained from the participants using a voluntary informed consent form prepared in accordance with the Declaration of Helsinki.

## Results

The mean age of the women in this study was 29.35 ± 5.55 years (range:18-45) and their mean years of education was 9.74±5.19 (min:0 max:16), mean number of pregnancies was 2.66 ± 1.78 (range:1-10), and mean number of births was 1.39± 1.56 (range:0-7). Other socio-demographic and obstetric characteristics of the participants are shown in Table 1.

**Table 1:** Sociodemographic characteristics of the participants (N = 363)

Variable		n	%
Age	18–23	65	17.9
	24–29	120	33.1
	30–35	127	35.0
	≥36	51	14.0

Education Level	No formal education	39	10.7
	Elementary school	67	18.5
	Middle school	68	18.7
	High school	76	20.9
	University	113	31.1

Place of Residence	Urban area	248	68.3
	Suburban area	84	23.1
	Rural area	31	8.6

Economic Status	Income < expenses	111	30.6
	Income = expenses	213	58.7
	Income > expenses	39	10.7

Employment Status	Working	78	21.5
	Not working	285	78.5

Gravidity	1	120	33.1
	2	84	23.1
	3	70	19.3
	≥ 4	89	24.5

Weeks of Gestation	14–27	88	24.2
	≥ 28	275	75.8

Miscarriage History	Yes	93	25.6
	No	270	74.4

Parity	0	134	36.9
	1	93	25.6
	2	67	18.5
	≥ 3	69	19.0

Planned Mode of Delivery	Vaginal birth	225	62.0
	Cesarean delivery	138	38.0

Social Support during Pandemic	Yes	267	73.6
	No	96	26.4

To what degree has the pandemic affected your pregnancy?	Very much	98	27.0
	Somewhat	227	62.5
	Not at all	38	10.5

**Total**		**363**	**100.0**

The participants' mean FCV-19S score was 19.03 ± 5.65 (range: 7-35). In the TPDS, the mean total score was 19.97 ± 7.97 (range: 1- 45), the mean negative affect subscale score was 15.13 ± 8.13 (range: 0-33), and the mean partner involvement subscale score was 4.83 ± 3.53 (range: 0-15). Based on the TPDS total score cut-off value (≥ 28), we determined that 69 (19.0%) of the women were at risk of pregnancy distress. In the TPDS subscales, 80 (22.0%) of the women were at risk according to the negative affect cut-off value (≥ 22) and 47 (12.9%) were at risk according to the partner involvement cut-off score (≥ 10).

A comparison of FCV-19S scores according to some descriptive characteristics is shown in Table 2. FCV-19S scores were significantly higher among participants who reported being highly affected by the COVID-19 pandemic compared to those who reported being somewhat or not at all affected, and were also higher among participants who reported being somewhat affected compared to those who were not affected at all (p < 0.05).

**Table 2:** Comparison of the participants' Fear of COVID-19 Scale scores according to selected characteristics

Variable		Mean	SD	Statistics	p	Bonferroni
Education Level	No formal education	18.08	5.59	1.719***	0.145	
	Elementary school	20.01	5.39			
	Middle school	19.62	5.84			
	High school	19.42	5.17			
	University	18.18	5.94			

Place of Residence	Urban area	19.15	5.84	1.942***	0.145	
	Suburban area	19.40	5.13			
	Rural area	17.16	5.22			

Economic Status	Income < expenses	19.20	5.10	1.999***	0.137	
	Income = expenses	19.26	5.84			
	Income > expenses	17.33	5.94			

Employment Status	Working	18.44	4.99	-1.058**	0.291	
	Not working	19.20	5.82			

Weeks of Gestation	14–27	18.72	6.05	-0.588**	0.557	
	≥28	19.13	5.52			

Planned Mode of Delivery	Vaginal birth	18.75	5.48	0.617**	0.538	
	Cesarean delivery	19.50	5.90			

Social Support during Pandemic	Yes	19.37	5.41	-1.227**	0.221	
	No	18.11	6.21			

To what degree has the pandemic affected your pregnancy?	Very much (1)	21.26	5.60	20.503***	0.000*	1>2, 1>3, 2>3
	Somewhat (2)	18.79	5.22			
	Not at all (3)	14.79	5.65			

Do you have sufficient knowledge about COVID-19?	Yes	18.92	5.91	0.432***	0.649	
	No	19.52	4.82			
	Somewhat	18.80	5.85			

* p < 0.05

** Independent t test

*** One-way analysis of variance

The comparison of TPDS total mean scores according to selected participant characteristics is shown in Table 3. TPDS total scores were significantly higher among participants who were not working, whose income was less than their expenses, who were in the third trimester of pregnancy (28 weeks gestation or later), and those who reported that they were highly affected by the COVID-19 (p < 0.05).

**Table 3:** Comparison of the participants' TPDS total scores according to selected characteristics

Variable		Mean	SD	Statistics	p	Bonferroni
Education Level	No formal education (1)	18.90	7.50	2.485***	**0.043** *	**2>5**
	Elementary school (2)	22.03	8.48			
	Middle school (3)	20.35	7.36			
	High school (4)	20.61	7.79			
	University (5)	18.48	8.08			

Place of Residence	Urban area	19.71	8.03	0.473***	0.624	
	Suburban area	20.45	8.00			
	Rural area	20.84	7.50			

Economic Status	Income < expenses (1)	22.25	8.68	9.161***	**0.000** *	**1>2, 1>3**
	Income = expenses (2)	19.43	7.62			
	Income > expenses (3)	16.49	5.81			

Employment Status	Working	18.33	8.16	-2.062**	**0.040** *	
	Not working	20.42	7.87			

Weeks of Gestation	14–27	17.38	7.34	-3.556**	**0.000** *	
	≥28	20.80	7.99			

Planned Mode of Delivery	Vaginal birth	19.64	8.10	-1.009**	0.313	
	Cesarean delivery	20.51	7.76			

**Social Support** **during Pandemic**	Yes	19.95	8.37	-0.095**	0.924	
	No	20.04	6.79			

To what degree has the pandemic affected your pregnancy?	Very much (1)	22.42	8.07	9.046**	**0.000** *	**1>2, 1>3**
	Somewhat (2)	19.51	7.70			
	Not at all (3)	16.47	7.72			

Do you have sufficient knowledge about COVID-19?	Yes	19.84	8.29	0.431***	0.650	
	No	20.65	7.37			
	Somewhat	19.61	7.92			

* p < 0.05

** Independent t test

*** One-way analysis of variance

Table 4 shows the results of our logistic regression analysis of factors associated with the participants' fear of COVID-19 and pregnancy distress. According to the results of the regression analysis, the models were statistically significant (p < 0.05). In model 1, Age (t = -3.256, p < 0.05), years of education (t = -4.375, p < 0.05), and number of births (t = 2.059, p < 0.05) were found to be significantly associated with fear of COVID-19. FCV-19S scores decreased by 0.217 with each additional year of age (β = -0.217), decreased by 0.272 with each additional year of education (β = -0.272), and increased by 0.502 with each additional birth (β = 0.502). We observed that 12.2% of the generated model was explained ( = 0.122). In model 2, FCV-19S score (t = 6.246, p < 0.05) and age (t = -2.442, p < 0.05) were significantly associated with TPDS Negative Affect subscale score. Negative Affect score increased by 0.469 with each 1-unit increase in FCV-19S score (β = 0.469) and decreased by 0.234 with each additional year of age (β = 0.234). Fifteen percent of the generated model was explained ( = 0.150). In model 3, FCV-19S score (t = -3.020, p < 0.05) and age (t = 4.423, p < 0.05) were significantly associated with TPDS Partner Involvement score. Partner Involvement score decreased by 0.100 with each 1-unit increase in FCV-19S score (β = -0.100) and increased with each additional year of age (β = 0.187). Again, 12.2% of the model was explained ( = 0.122). In model 4, FCV-19S score (t = 4.813, p < 0.05) was significantly associated with TPDS total score. TPDS total score increased by 0.047 with each 1-unit increase in FCV-19S (β = -0.047). This explained 7.8% of the model ( = 0.078)

**Table 4:** Logistic regression analysis of factors associated with fear of COVID-19 and Pregnancy Distress

Model	Dependent Variable	Independent Variable	β	Standard Error	Beta	t	p	F	Model (p)	R2
1	FCV-19S Score	Constant	27.784	1.748	-	15.893	**0.000** *	9.934	**0.000** *	0.122
		Age	-0.217	0.067	-0.213	-3.256	**0.001** *			
		Years of Education	-0.272	0.062	-0.251	-4.375	**0.000** *			
		Gravidity	-0.225	0.202	-0.071	-1.112	0.267			
		Parity	0.502	0.244	0.139	2.059	**0.040** *			
		Number of Miscarriages	0.422	0.414	0.057	1.019	0.309			

2	TPDS– Negative Affect Score	Constant	13.048	3.241	-	4.026	**0.000** *	10.467	**0.000** *	0.150
		FCV-19S Score	0.469	0.075	0.326	6.246	**0.000** *			
		Age	-0.234	0.096	-0.160	-2.442	**0.015** *			
		Years of Education	-0.018	0.091	-0.012	-0.202	0.840			
		Gravidity	0.257	0.287	0.057	0.897	0.370			
		Parity	0.023	0.348	0.004	0.067	0.947			
		Number of Miscarriages	-1.030	0.598	-0.104	-1.362	0.055			

3	TPDS–Partner Involvement Score	Constant	1.429	1.432	-	0.998	0.319	8.270	**0.000** *	0.122
		FCV-19S Score	-0.100	0.033	-0.160	-3.020	**0.003** *			
		Age	0.187	0.042	0.294	4.423	**0.000** *			
		Years of Education	-0.015	0.040	-0.022	-0.379	0.705			
		Gravidity	-0.026	0.127	-0.013	-0.202	0.840			
		Parity	-0.051	0.154	-0.022	-0.331	0.741			
		Number of Miscarriages	0.284	0.260	0.061	1.093	0.275			

4	TPDS– Total score	Constant	14.477	3.307	-	4.377	**0.000** *	5.026	**0.000** *	0.078
		FCV-19S Score	0.369	0.077	0.261	4.813	**0.000** *			
		Age	-0.047	0.098	-0.033	-0.478	0.633			
		Years of Education	-0.034	0.093	-0.022	-0.362	0.717			
		Gravidity	0.232	0.293	0.052	0.792	0.429			
		Parity	-0.028	0.355	-0.005	-0.078	0.938			
		Number of Miscarriages	-1.046	0.600	-0.099	-1.744	0.082			

* p < 0.05

There was a statistically significant positive correlation between FCV-19S scores and TPDS total and subscale scores (r=0.263, p<0.05; Table 5).

**Table 5:** Pearson correlation analysis between FCV-19S score and TPDS scores

	1	2	3	4
1. TPDS–Negative Affect	1.000	-0.263	0.904	0.341
P	-	0.000*	0.000*	0.000*
2. TPDS–Partner Involvement	1.000	0.175	-0.191
P		-	0.001*	0.000*
3. TPDS–Total			1.000	0.263
P			-	0.000*
4. FCV-19S				1.000
P				-

* p < 0.05

## Discussion

### Fear of COVID-19 and associated factors

Pregnancy is a natural but complex period in life that requires adaptation to physiological, psychological, and social changes. On the other hand, pregnancy is a time in which the line between health and disease is thinnest^33^. Infectious diseases are significant factors associated with maternal and fetal mortality and morbidity and can be a source of fear for pregnant women 34. This is also the case with COVID-19. Tesfamicheal et al. (2021) reported that 51% of pregnant women in Ethiopia were afraid of COVID-1912. In another study conducted in Ethiopia, pregnant women had a mean FCV-19S score of 27.1 ± 5.2 and 43.8% were afraid of COVID-19 35. In two separate studies conducted in Pakistan, fear of COVID-19 was common among women (60% to 84.6%) 13,14. On the other hand, two studies conducted in Iran reported mean FCV-19S scores of 22.5 ± 5.9 and 22.29 ± 7.0 in pregnant women^36^,^37^. In a Japanese study by Asai et al. (2021), pregnant women had a mean FCV-19S score of 22.96 ± 5.69 10. The pregnant women in our study had a mean FCV-19S of 19.03 ± 5.65, indicating moderate fear of COVID-19. The level of fear of COVID-19 in our study was lower but comparable to rates reported in Japan and Iran but substantially lower than reported in Ethiopia and Pakistan. This may be related to differences in level of development and culture between countries.

The most important source of fear is the unknown. The COVID-19 pandemic also continues to generate fear and anxiety due to its many unknowns 38. In the regression analysis conducted in this study, we determined that higher age and years of education were associated with lower fear of COVID-19. On the other hand, Nausheen et al. (2020) determined that age and years of education had no effect on fear of COVID-19 among the pregnant women in their study^14^. In contrast, a study conducted in a non-pregnant population showed that fear of COVID-19 was lower in individuals with high education level but increased with age^39^. Similarly, in our study we presume that higher education level reduced the level of fear in pregnant women by helping them better understand the pandemic and increase their self-efficacy. On the other hand, since the participants in this study were all young women of reproductive age, there was not as great a difference between the age groups as in the study by Cori et al. (2020). Therefore, as with years of education, we also attribute the lower level of fear in older pregnant women to higher levels of knowledge and self-efficacy.

Parity is considered a factor that affects mental health during pregnancy^9^,^23^,^40^. We also observed in this study that higher parity was associated with a greater fear of COVID-19. This may be related to the physical effects of pregnancy and childbirth on the women and/or the increased care burden with more children. In this study, pregnant women who said they were highly affected by the COVID-19 pandemic had higher fear and distress than those who stated that the pandemic had little or no effect on them. This finding shows that even simple statements about fear of COVID-19 made by pregnant women effectively reflect their mental state. Therefore, it is beneficial to provide pregnant women opportunities to express themselves during antenatal visits.

Our results indicated that pregnant women's fear of COVID-19 was not associated with their place of residence, economic status, employment status, gravidity, history of miscarriage, trimester of pregnancy, planned mode of delivery, presence of social support, or perceived need for professional support. Similarly, Nausheen et al. (2020) detected no relationship between fear of COVID-19 and gravidity, trimester, income level, or employment status in their 2020 study 14.

### Pregnancy distress

The prevalence of mental health problems during pregnancy under normal circumstances varies between and even within countries 41. For example, in three pre-COVID-19 studies conducted in Turkey, the mean TPDS scores of pregnant women ranged from 11.63 ± 6.40 to 23.66 ± 7.48 and the proportion of pregnant women at risk of distress was between 9.6% and 33% 22,23,42. In a study conducted in Africa, the prevalence of pregnancy distress was reported to be 38.6%, while in a study conducted in the United States, 21.2% of women had pregnancy distress^20^,^43^.

The prevalence of mental health problems in pregnant women during the COVID-19 pandemic, which can be described as one of the biggest global disasters of the century, also varied substantially between countries. In a study conducted in Turkey, the average TPDS score was 24.09 ± 7.29 and the risk of pregnancy distress was 37%^24^. In two similar studies conducted in Turkey, the prevalence of anxiety among pregnant women was 29.6% and 64.5% 25,44. A study conducted in Indonesia showed that 42% of pregnant women had moderate anxiety and 32% had moderate to high anxiety 7. In a study conducted in Colombia, anxious symptoms were identified in 50.4% of pregnant women44. The prevalence of pregnancy-related anxiety was reported to be 21% in Iran and between 6.8% and 36.7% in three studies conducted in China^8^,^9^,^45^,^46^. A three-centre study involving Ireland, the United States, and the United Kingdom revealed high pregnancy-related stress (13.89 ± 5.37) and fear of COVID-19 (7.14 ± 2.84)^47^.

The pregnant women in our study had a mean TPDS score of 19.97 ± 7.97 and 19.0% were at risk of distress according to the cut-off score. Compared to the literature cited above, the participants in this study had a slightly higher level of distress than was reported in Turkey before the COVID-19 pandemic. However, their distress level was considerably lower than rates in previous reports from Turkey and other countries during the pandemic, except for the study by Zhou et al. (2021)^8^. We suspect that the very positive perception of fertility in the province where the study was conducted contributed to the low distress level of these pregnant women^27^. It is also noteworthy that most of the studies cited above were conducted by telephone or using web-based methods. Ultimately, participation in social media or telephone surveys requires having and actively using technological resources. This leads to the supposition that the results of such studies do not reflect the mental state of pregnant women who lack this access or inclination. Our study was conducted via face-to-face interviews, which enabled us to evaluation pregnancy distress in women from all segments of society.

The positive association between fear of COVID-19 and TPDS total and Negative Affect subscale scores in our regression analysis and the significant correlation between the two scales observed in this study indicate that fear of COVID-19 is an important factor associated with greater pregnancy distress. Fear of COVID-19 was previously shown to cause anxiety disorder in pregnant women in one study, while another showed that COVID-19-related anxiety caused prenatal distress 9,13. Our findings support these two studies. Based on our results and those in the relevant literature, we conclude that for pregnant women, the negative conditions induced by the COVID-19 pandemic first turn into fear and then distress in the presence of other predisposing factors. In addition, it is noteworthy that regression analysis determined that greater fear of COVID-19 was positively associated with the Partner Involvement subscale of the TPDS. This shows that pregnant women who are afraid of COVID-19 are supported by their partners.

In the literature, some studies have shown that low maternal age is a predisposing factor for prenatal distress, while others suggested prenatal distress is positively associated with advanced maternal age^22^, ^48^–^51^. In contrast, there are also studies showing that age is not related to prenatal distress 52,53. The results of the present study demonstrate that the mitigating effect of the experience and self-efficacy gained with age on negative affect facilitates stress management in pregnant women. On the other hand, the negative relationship between maternal age increase and partner involvement suggests the effect of changes in spousal dynamics over time.

It has been reported that higher level of education corresponds to a greater ability for pregnant women to cope with stress^54^,^55^. The results of studies investigating the effects of education on the mental health of pregnant women during the COVID-19 pandemic vary. Wang et al. (2021) reported that high education level was associated with depression in pregnant women^45^. Ge et al. (2021) stated that low education contributes to the development of anxiety46. In this study, low education level was found to be associated with pregnancy distress, but it was not a significant predictor of pregnancy distress in logistic regression analysis.

As with education, employment status and income level directly affect women's health; pregnant women with low income were found to have higher levels of anxiety, depression, and concern^56^,^57^,^58^. Unfortunately, the isolation and closure orders implemented to reduce the spread of COVID-19 have affected the poverty of women more deeply, and studies have reflected the impact of this economic crisis on antenatal stress 46, 59. High distress scores among the low income level and the non-working pregnant women in this study are consistent with the literature. The second trimester of pregnancy is a stable period in which pregnancy-related disorders regress and adaptation to pregnancy is high. In contrast, the third trimester is a riskier period in terms of mental health since concerns about birth, the postpartum period, and infant care intensify during this time^60^. Ge et al. (2021) reported that women in the first and second trimester of pregnancy had higher anxiety scores than those in the second trimester^46^. We also observed a higher level of pregnancy distress in the third trimester, consistent with the literature.

Studies have reported different relationships between pregnancy distress and women's gravidity and parity. For example, Dündar et al. (2019) reported that the prevalence of pregnancy distress was higher in women with more pregnancies and more children^23^. However, a study by Lebel et al. (2020) during the COVID-19 pandemic indicated that distress levels were higher in nulliparous women^40^. Hamzehgardeshi et al. (2021) also reported that number of pregnancies was associated with distress level^9^. Contrary to these examples, Koyucu et al. (2020) and Taşlar and Kocatepe (2018) observed no difference in distress level according to parity, but reported that distress was associated with different factors in the parity groups. Our finding that gravidity and parity were not independent factors in pregnancy distress is consistent with these two studies 61,62. We also observed that number of miscarriages, level of knowledge about COVID-19, place of residence, presence of social support, and planned mode of delivery were not associated with pregnancy distress. This contradicts the results of Hamzehgardeshi et al. (2021), who determined that COVID-19 knowledge and social support affected levels of distress in pregnant women^9^.

## Conclusion

The pregnant women in this study had moderate fear of COVID-19. Older age and higher education were factors associated with lower fear of COVID-19, while higher parity was associated with greater fear. When compared with the literature, the prevalence of pregnancy distress in this study was slightly higher than before COVID-19 but quite low compared to other studies conducted during the pandemic. Factors related to pregnancy distress in this study were fear of COVID-19, education level, income level, working status, and trimester of pregnancy. Among these factors fear of COVID-19 was negatively associated with pregnancy distress. In addition, we determined that older maternal age was positively associated with negative affect and negatively associated with partner involvement.
